# *Arabidopsis thaliana* Yellow Stripe1-Like4 and Yellow Stripe1-Like6 localize to internal cellular membranes and are involved in metal ion homeostasis

**DOI:** 10.3389/fpls.2013.00283

**Published:** 2013-07-26

**Authors:** S. S. Conte, H. H. Chu, D. Chan-Rodriguez, T. Punshon, K. A. Vasques, D. E. Salt, E. L. Walker

**Affiliations:** ^1^Biology, University of MassachusettsAmherst, MA, USA; ^2^Biology, Dartmouth CollegeHanover, NH, USA; ^3^Plant Biology Graduate Program, University of MassachusettsAmherst, MA, USA; ^4^Biogen-IdecCambridge, MA, USA; ^5^Institute of Biological and Environmental Sciences, University of AberdeenAberdeen, Scotland

**Keywords:** nickel, nicotianamine, yellow stripe-like, metal transporters, manganese, iron, tonoplast, endomembrane

## Abstract

Several members of the Yellow Stripe1-Like (YSL) family of transporter proteins are able to transport metal-nicotianamine (NA) complexes. Substantial progress has been made in understanding the roles of the Arabidopsis YSLs that are most closely related to the founding member of the family, ZmYS1 (e.g., AtYSL1, AtYSL2 and AtYSL3), but there is little information concerning members of the other two well-conserved YSL clades. Here, we provide evidence that AtYSL4 and AtYSL6, which are the only genes in Arabidopsis belong to YSL Group II, are localized to vacuole membranes and to internal membranes resembling endoplasmic reticulum. Both single and double mutants for YSL4 and YSL6 were rigorously analyzed, and have surprisingly mild phenotypes, in spite of the strong and wide-ranging expression of YSL6. However, in the presence of toxic levels of Mn and Ni, plants with mutations in YSL4 and YSL6 and plants overexpressing GFP-tagged YSL6 showed growth defects, indicating a role for these transporters in heavy metal stress responses.

## Introduction

Members of the Yellow Stripe Like (YSL) family of transporters are required for normal iron, zinc, manganese and copper movement in both vegetative and reproductive tissues (DiDonato et al., 2004; Koike et al., 2004; Roberts et al., 2004; Schaaf et al., 2004; Murata et al., 2006; Gendre et al., 2007; Aoyama et al., 2009; Curie et al., 2009; Inoue et al., 2009; Lee et al., 2009; Chu et al., 2010; Sasaki et al., 2011; Zheng et al., 2011, 2012). Work on the YSL family started with cloning of the maize *Yellow stripe1* (*ZmYS1*) gene (Curie et al., 2001). Transport through YS1 is the primary route by which roots of grasses take up iron from the soil. The grasses, a group that includes most of the world's staple grains (e.g., rice, wheat and corn), use a chelation strategy for primary iron uptake. In response to iron starvation, grasses secrete phytosiderophores (PS), which are non-proteinogenic amino acid derivatives of the mugineic acid (MA) family that form stable chelates with Fe(III) (Tagaki et al., 1984). This accomplishes solubilization of the otherwise nearly insoluble soil iron. The *ZmYS1* gene encodes a protein that is distantly related to the Oligopeptide Transporter (OPT) family of proteins (Curie et al., 2001; Yen et al., 2001) and functionally complements yeast strains that are defective in iron uptake when grown on medium containing Fe(III)-PS complexes.

Although non-grass plant species neither synthesize nor efficiently use PS, Yellow Stripe1-Like (YSL) proteins are found in monocots and dicots, as well as gymnosperms, ferns and mosses. The major physiological role of the YSLs appears to be in the movement of metals bound to the ubiquitous plant metal chelator, nicotianamine (NA). It has been well-established that several YSL proteins are indeed able to transport metal-NA complexes (DiDonato et al., 2004; Koike et al., 2004; Roberts et al., 2004; Schaaf et al., 2004; Le Jean et al., 2005; Murata et al., 2006; Gendre et al., 2007; Harada et al., 2007) and that NA is essential for long-distance transport of metals throughout the plant body (Schuler et al., 2012). NA is capable of forming complexes with manganese (Mn), Fe(II), cobalt (Co), zinc (Zn), nickel (Ni) and copper (Cu) in increasing order of affinity (Anderegg and Ripperger, 1989). However, little is known about the role of NA in intracellular transport of metals. Pich et al. used an NA-specific antibody to localize NA in the vacuoles of Fe-loaded tomato cells, which suggests a role for NA in the vacuolar storage of excess Fe (Pich et al., 2001). Recently, Haydon et al. showed that overexpression of the transporter Zinc Induced Facilitator1 (ZIF1) caused an increase in vacuolar NA in roots with a concomitant increase in vacuolar Zn, thus implicating NA in the vacuolar storage of Zn (Haydon et al., 2012). Because YSLs are known metal-NA transport proteins, it is reasonable that members of the YSL family could participate in the intracellular transport of NA.

Now that several plant genomes have been sequenced, it is clear that higher plants possess four distinct, well-conserved groups of YSL proteins, and that one of these is unique to grass species (Curie et al., 2009; Yordem et al., 2011). Substantial progress has been made in understanding the roles of the YSLs that are most closely related to ZmYS1 [e.g., AtYSL1, AtYSL2 and AtYSL3 (DiDonato et al., 2004; Waters et al., 2006; Chu et al., 2010) and OsYSL2 (Koike et al., 2004; Inoue et al., 2006; Ishimaru et al., 2010)], but there is little information concerning members of the other two conserved YSL clades. The most basal clade of the YSL family tree contains YSLs from the moss *Physcomitrella patens*, the lycophyte *Selaginella moellendorffii*, OsYSL5 and 6 from rice, HvYSL5 from barley and AtYSL4 and 6 from Arabidopsis (Yordem et al., 2011; Zheng et al., 2011). Details about the members of this group have just begun to emerge. Interestingly, Jaquinod et al. identified AtYSL4 and AtYSL6 as members of the tonoplast proteome (Jaquinod et al., 2007). The barley protein, HvYSL5, was found to localize either to vesicles or the tonoplast based on bombardment of onion skin cells with the ORF of *HvYSL5* fused to *smGFP* (Zheng et al., 2011). Localization of the rice protein, OsYSL6, was inconclusive; bombardment experiments indicated that regardless of whether GFP was fused to the N- or C-terminus, the GFP signal appeared cytoplasmic (Sasaki et al., 2011). Very recently, Divol et al. (2013) used immunofluorescence imaging to conclude that the Arabidopsis AtYSL4 and AtYSL6 proteins are located in plastids. Taken together, these localization data suggest that YSLs in the most basal clade may play roles in the intracellular transport of metal chelates.

In this study, we investigated the role of the two closely related Arabidopsis group II YSL genes, *AtYSL4* (AT5G41000) and *AtYSL6* (AT3G27020). *AtYSL4* and *AtYSL6* mRNAs are abundantly expressed in Arabidopsis, especially during seed germination, but surprisingly, neither null single (*ysl4* and *ysl6*) mutants nor the *ysl4ysl6* double mutant exhibits strong visible phenotypes. The levels of several transition metals are modestly perturbed in both single and double mutants, but localization of metals in the seeds is unaltered. Using transient transformation of GFP fusions into poorly-conserved cytosolic domains of the proteins, we observed a pattern that is consistent with localization of AtYSL4 and AtYSL6 to vacuolar membranes within the cell. When the same YSL6mid GFP construct was stably transformed into Arabidopsis, we observed a pattern of fluorescence consistent with localization to internal membranes resembling the endoplasmic reticulum. Loss of *AtYSL4*, *AtYSL6* or both confers almost no measurable phenotypic change other than an alteration in the plants' sensitivity to excess manganese. When excess Mn and Ni are added to iron deficient medium, alterations in root growth occur in both mutant and overexpressing plants. Taken together, these data indicate a role for AtYSL4 and AtYSL6 in intracellular transport of metal-NA complexes.

## Materials and methods

### Plant growth

#### Plate-grown plants

Seeds were sterilized in eppendorf tubes (1.5 mL) or a 15 mL falcon tube depending on the quantity and suspected level of contamination of the seeds. Seeds were soaked in 70% ethanol and 0.05% Triton X-100 for 10 min with occasional vortexing, then three times with 100% ethanol. Seeds were gently placed onto sterile Whatman paper and allowed to dry. They were then either imbibed in sterile 0.1% agarose at 4°C for 3–5 days, or were plated, wrapped in foil, and stored at 4°C for 3 days prior to transfer to the growth chamber Plants were grown on sterile 1X MS agar medium with or without antibiotics. Plates were placed in an upright position so that the roots grew along the surface rather than inside the agar, which allowed for easy transfer. Plates were placed in an incubator at 22°C with 16 h of light and 8 h of darkness.

#### Soil-grown plants

Seeds imbibed in distilled water at 4C in the dark for 3 days, and then were sown directly onto Metro-Mix (Sungro Horticulture) treated with Gnatrol (Valent Biosciences) to control fungus gnats. Growth chamber conditions were the same as for plate-grown plants.

### *AtYSL4* and *AtYSL6* expression analysis using RT-PCR

Plant parts were ground in 1.5 mL tubes using RNase-free disposable pestles (VWR, Batavia, Illinois). Total RNA was isolated using the RNeasy plant mini kit (Qiagen, Valencia, CA), followed by DNase treatment using the DNA-free kit (Ambion, Austin, Texas). The total RNA concentration was quantified using a spectrophotometer (260 nm; Beckman DU640B, Fullerton, CA), and confirmation of RNA quality was performed by visualization on a 1X TBE gel stained with ethidium bromide.

### Quantitative RT-PCR

RNA isolation and RT reactions were performed as described (Waters et al., 2006). Quantitative real-time PCR was also performed as described (Chu et al., 2010). YSL4- and YSL6-specific primer sequences were as follows: for AtYSL4, oAtYSL4.qPCR Fw (5′-TCGTTCCACTTCGCAAGGTGATG-3′) and oAtYSL4.qPCR Rev (5′-ACATTGCTGTAGCGGTTCCACTG-3′); for AtYSL6, oAtYSL6.qPCR Fw (5′-TCGTTCCGTTACGCAAGGTG-3′) and oAtYSL6.qPCR Rev (5′-AGCTCCAGTGTTGGTGTGGAAG-3′).

### Promoter::GUS constructs

The *AtYSL4* promoter region containing 785 bp upstream of the *AtYSL4* initiating ATG was cloned to create a C-terminal translational fusion to the GUS reporter gene. The *AtYSL6* promoter region containing 601 bp upstream of the *AtYSL6* initiating ATG was cloned to create a C-terminal translational fusion to the GUS reporter gene. The *AtYSL4p*::GUS and *AtYSL6p*::GUS constructs were then stably introduced into Arabidopsis using the floral dip method (Clough and Bent, 1998). T1 seeds were germinated on 1X MS medium with 50 μM kanamycin to select for transformants, and seedlings were transferred to soil 7 days after germination.

### Preparation of samples for mineral analysis

For soil-grown plants, leaf samples were collected 20 days after sowing and dried in an oven at 60°C. ICP-MS was performed as described previously (Lahner et al., 2003).

### Construction of GFP-mid tagged proteins

*AtYSL4* (At5g41000) and *AtYSL6* (At3g27020) cDNAs were amplified by RT-PCR using Platinum *Taq* DNA Polymerase High Fidelity (Invitrogen, Carlsbad, CA). *AtYSL4* cDNA was amplified using the primers 5′-TCTGAGAGTGAGAGGAATCACTGAAAA-3′ and 5′-GTCTCGGATGGTCTAAAGTACATACAAATGGGTG-3′. *AtYSL6* cDNA was amplified using the primers 5′-GCTAAAACATGGGGACGGAGATCCC-3′ and 5′-CTCTCTCTTGCTGAGGACGGTCCAAA-3′. *AtYSL4* and *AtYSL6* cDNA were then cloned into the Gateway vector pCR8/GW/TOPO (Invitrogen, Carlsbad, CA). Using the “megaprimer” method, *smGFP* (Davis and Vierstra, 1998) was incorporated into *AtYSL4* between position 1053 and 1054 in the cDNA (corresponding to amino acid position 351) and *AtYSL6* between position 1086 and 1087 in the cDNA (corresponding to amino acid position 362). Based on protein structure predictions, these positions correspond to extracellular loops that are weakly conserved among YSLs. The primers 5′-GCAACAAAAGCTCCAGACAAGGGATCCAAGGAGATATAACAATGA-3′ and 5′-GTCGGTAAAGACAGGTAGGTTGTGTTTGTATAGTTCATCCATGCCAT-3′ (for *AtYSL4*), and 5′-CAATCTACCCATTGTTACCGACGGGATCCAAGGAGATATAACAATGA-3′ and 5′-GAAGCTTCACTGTCATCTACACCTTTGTATAGTTCATCCATGCCAT-3′ (for *AtYSL6*) were used to amplify the plasmid psmGFP (CD3-326, available from ABRC) to create megaprimers containing the smGFP sequence flanked by specific YSL sequences. These megaprimers were gel purified and used in a modified site-directed mutagenesis protocol to introduce the smGFP sequence into the AtYSL4 and AtYSL6 clones described above. This was accomplished using 440 ng of purified megaprimers, 50 ng of target vector (either *YSL4* or *YSL6* cDNA in pCR8/GW/TOPO, described above), 0.6 mM dNTP mix, 1X Phusion HF buffer (New England Biolabs, Ipswich, MA), 0.25 μl of Phusion enzyme (New England Biolabs, Ipswich, MA), and the following PCR conditions: an initial denaturation at 98°C for 2 min, followed by 18 cycles of 98°C for 50 s, 50°C for 50 s, and 72°C for 5 min, followed by a final elongation of 72°C for 7 min. After DpnI digestion to remove the methylated parent plasmid, 2 ml of each reaction was used to transform TOP10™ chemically competent *E. coli* (Invitrogen, Carlsbad, CA). Positive clones were verified by restriction digestion and sequencing to ensure incorporation of the full smGFP sequence. An LR recombination reaction was then performed to transfer *AtYSL4-GFPmid* and *AtYSL6-GFPmid* into the vector pB7WG2 (Karimi et al., 2002) for subsequent transient expression in Arabidopsis protoplasts in addition to stable expression in Arabidopsis plants.

### Protoplast isolation and transformation

Protoplasts were isolated using the Tape-Arabidopsis Sandwich method (Wu et al., 2009). Transformations were carried out using 5 × 10^4^ protoplasts in 200 μL of MMg solution (0.4 M mannitol, 15 mM MgCl_2_, 4 mM MES pH 5.7) mixed with 30 μg of plasmid DNA at room temperature. An equal volume of freshly prepared transformation solution [40% w/v PEG (MW 4000), 0.1 M CaCl_2_, 0.2 M mannitol] was added and allowed to incubate for 7 min. Protoplasts were then carefully washed three times with modified W5 solution (154 mM NaCl, 125 mM CaCl_2_, 5 mM KCl, 5 mM glucose, 2 mM MES pH 5.7), resuspended in 200–500 μL modified W5, and incubated at room temperature in Mattek™ dishes for 16 to 24 h. *AtYSL4-GFPmid* or *AtYSL6-GFPmid* in pB7GW2 was co-transformed with CD3-976, which is available from TAIR (www.arabidopsis.org) and contains γ-TIP fused to mCherry.

### Staining of protoplasts with FM-464

The lipophilic dye FM-464 was applied to transformed protoplasts at a final concentration of 100 μM for 10 min on ice. The dye was then washed off, and protoplasts were incubated at RT for 1 h to allow the dye to penetrate the cells prior to confocal imaging.

### Plant transformation

All constructs to be used in plant transformation experiments were transferred to *Agrobacterium tumefaciens* GV3101 via electroporation. *Arabidopsis thaliana* plants were transformed by Agrobacterium-mediated transformation using the floral dip method (Clough and Bent, 1998). Primary transformants were selected by spraying with the herbicide Finale (Bayer, Research Triangle Park, NC). Individual progeny of selfed primary transformants were examined by confocal microscopy.

### Confocal microscopic analysis

Protoplasts were imaged using a Zeiss LSM 510 Meta Confocal System equipped with a 63x oil immersion objective. An argon 488 nm laser was used for excitation of GFP and a HeNe 543 laser was used for excitation of FM-464 and mCherry. Emission of GFP was collected between 505 and 530 nm and emission of FM-464 and mCherry was collected between 585 and 615 nm.

### Determination of root growth rate

Seeds were plated directly onto medium containing 1% phytoagar, and imbibed for 72 h at 4C in the dark. Plates were then transferred into the growth chamber and allowed to grow vertically. Photographs of the plates were taken every 24 h, and successive images were aligned using ImageJ. The difference in root length over successive 24 h periods was recorded and used to calculate the root growth rate in mm/h.

## Results

### Expression of *AtYSL4* and *AtYSL6*

**Figure 1:**
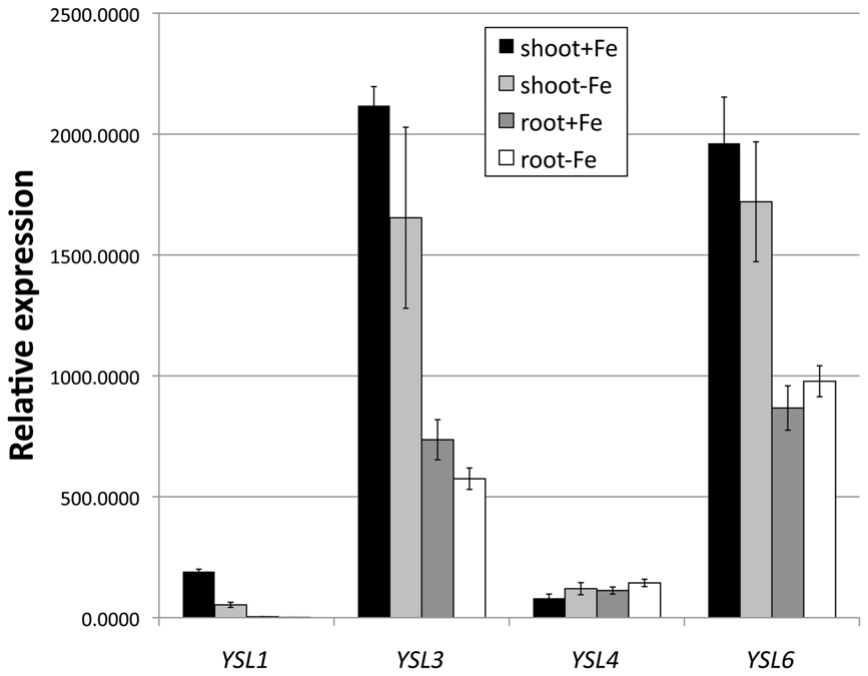
**Expression of AtYSL1, AtYSL3, AtYSL4 and AtYSL6 by quantitative RT-PCR**. Plants were grown on MS medium for 10 days and then transferred to MS and MS lacking iron for 5 days. mRNA levels were quantified in both shoots and roots.

To understand the level of expression of *AtYSL4* and *AtYSL6* relative to other members of the YSL family, we performed quantitative RT-PCR (Figure 1). *AtYSL6* is strongly expressed in both shoots and roots, and its mRNA is present at levels similar to *AtYSL3*. *AtYSL4* mRNA is expressed at lower levels, and it is expressed at similar levels in both shoots and roots. Because mRNA levels for *AtYSL1*, *AtYSL2*, and *AtYSL3* decrease in plants that have been grown under iron deficiency, we examined the level of *AtYSL4* and *AtYSL6* mRNA in iron deficient plants. The mRNA levels for *AtYSL4* and *AtYSL6* are not strongly affected by iron deficiency (Figure 1). Semi-quantitative RT-PCR (not shown) indicates that *AtYSL4* and *AtYSL6* mRNA levels are also not strongly affected by deficiency for other transition metals.

### Pattern of *AtYSL4* and *AtYSL6* expression

**Figure 2:**
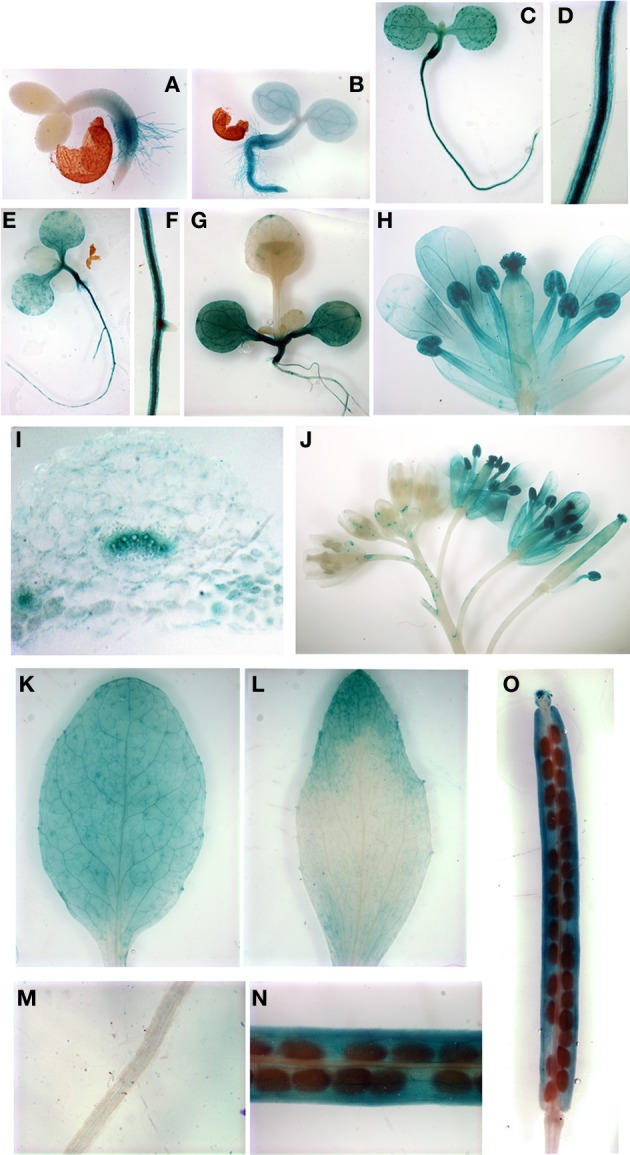
**Histochemical staining of AtYSL4 promoter-GUS reporter plants. (A)** Seedling at 48 h **(B)** Seedling at 72 h **(C)** Seedling at 5 days **(D)** Root of 5-day-old seedling **(E)** Seedling at 8 days **(F)** Root of 8-day-old seedling **(G)** Seedling at 11 days **(H)** Flower at 36 days **(I)** Cross-section of rosette leaf at 36 days **(J)** Flowers at 36 days illustrating mature and immature flowers **(K)** Rosette leaf at 37 days **(L)** Cauline leaf at 37 days **(M)** Root at 24 days **(N,O)** Siliques at 24 days.

In order to determine the cell type specific expression of *AtYSL4* and *AtYSL6*, we constructed β-glucuronidase (GUS) reporter constructs containing the promoter sequences of *AtYSL4* or *AtYSL6* fused in-frame to GUS (*AtYSL4p*:*GUS* and *AtYSL6p*:*GUS*). In germinating seedlings, *AtYSL4p:GUS* was expressed strongly in roots and root hairs at 48 h post-germination (Figure 2A); by 72 h, *AtYSL4p:GUS* expression had spread to cotyledons (Figures 2B,C) where it remained strong at 11 d post-germination (Figure 2E). Only minimal *AtYSL4p:GUS* expression was detected in true leaves (Figures 2E,G). Expression was also strong in flowers, especially older flowers, sepals and pollen (Figures 2H,J). A cross-section through a rosette leaf revealed that *AtYSL4p:GUS* expression was associated with xylem tissues (Figure 2I). In rosette leaves, expression was low and diffuse in interveinal regions (Figure 2K), and in cauline leaves, expression was restricted to older areas of the leaf (Figure 2L). In fruits, *AtYSL4p:GUS* was expressed most strongly in the veins of the siliques, with only weak expression in the developing seeds (Figures 2N,O). Expression was absent from roots at 24 d (Figure 2M).

**Figure 3:**
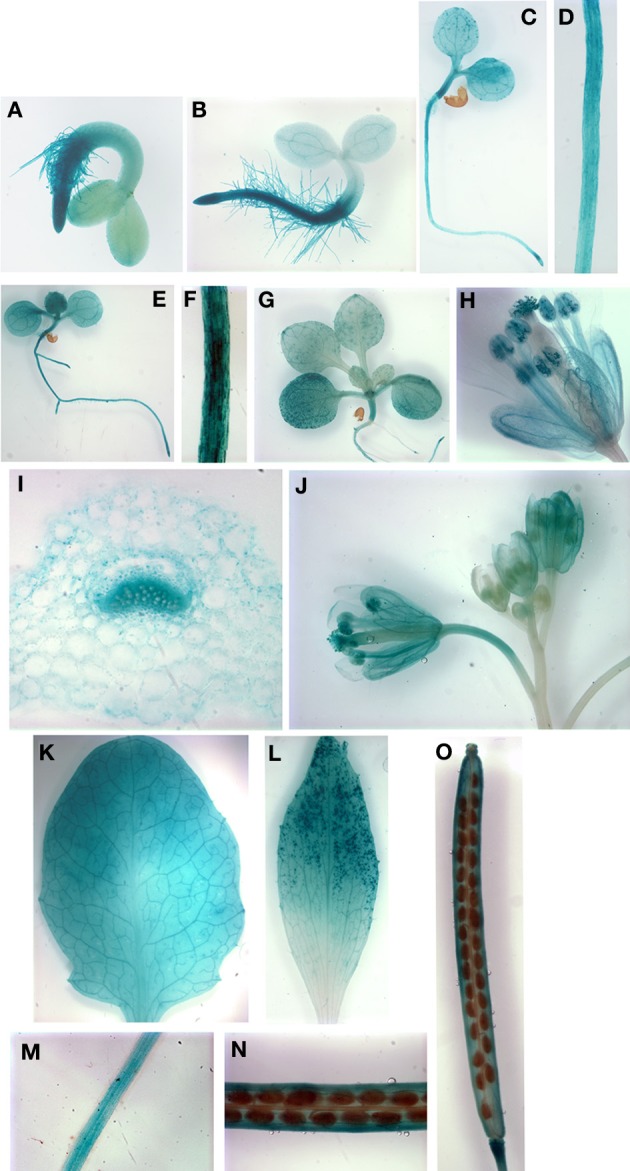
**Histochemical staining of AtYSL6 promoter-GUS reporter plants. (A)** Seedling at 48 h **(B)** Seedling at 72 h **(C)** Seedling at 5 days **(D)** Root of 5-day-old seedling **(E)** Seedling at 8 days **(F)** Root of 8-day-old seedling **(G)** Seedling at 11 days **(H)** Flower at 27 days **(I)** Cross-section of rosette leaf at 27 days **(J)** Flowers at 24 days illustrating mature and immature flowers **(K)** Rosette leaf at 27 days **(L)** Cauline leaf at 37 days **(M)** Root at 24 days **(N,O)** Siliques at 24 days.

*AtYSL6p:GUS* plants exhibited stronger staining in roots of very young developing seedlings as compared to *AtYSL4p:GUS* plants (Figures 3A,B), and by 5, 8 and 11 d, expression was evident in both cotyledons and true leaves (Figures 3C,E,G). Most of the cells in mature rosette leaves showed strong *AtYSL6p:GUS* expression, including very strong staining in the vasculature (Figure 3K). A rosette leaf cross section revealed that *AtYSL6p:GUS* expression was associated with xylem tissues (Figure 3I). Similar to *AtYSL4*, *AtYSL6* was expressed in the older regions of cauline leaves (Figure 3L), albeit more strongly than what was observed for *AtYSL4*. However, *AtYSL6* was not as strongly expressed in sepals and anther filaments, and expression was not completely restricted to older flowers (Figures 3H,J). *AtYSL6* expression in young roots was more diffuse than *AtYSL4*, and the expression was more closely associated with the root vasculature (compare Figures 2D,F with Figures 3D,F). Roots at 24 d expressed *AtYSL6p:GUS* in all tissue layers (Figure 3M). Similar to *AtYSL4*, *AtYSL6* was expressed in veins of siliques but was also evident to some extent in the developing seeds themselves (Figures 3N,O).

### Localization of AtYSL4 and AtYSL6 proteins

**Figure 4:**
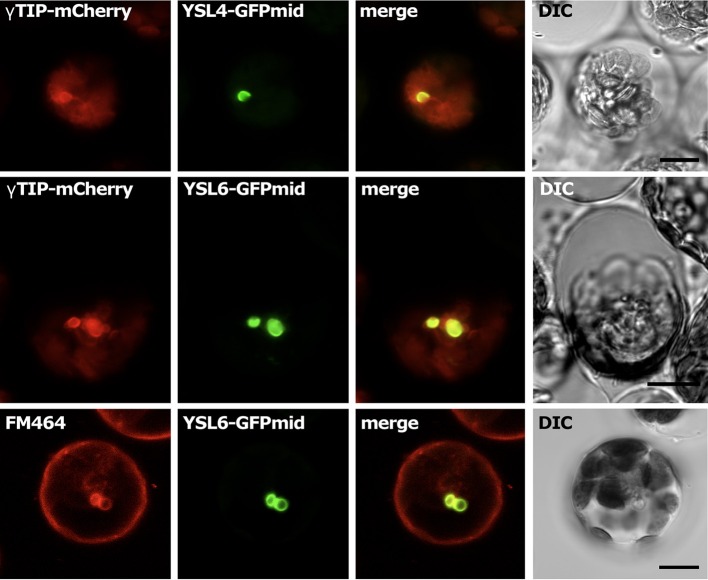
**Subcellular localization of AtYSL4 and AtYSL6 in protoplasts**. Each horizontal row of four images shows (left to right) red fluorescence, green fluorescence, merged red and green images, and differential interference contrast. **Top row:** protoplast co-transformed with *AtYSL4-GFPmid* and γ *-TIP-mCherry*. **Middle row:** protoplast co-transformed with *AtYSL6-GFPmid* and γ *-TIP-mCherry*. **Bottom row:** protoplast transformed with *AtYSL6-GFPmid* and stained with the membrane-selective dye FM-464. Scale bar = 10 um.

Although several YSLs are known to localize to the plasma membrane (DiDonato et al., 2004; Aoyama et al., 2009; Inoue et al., 2009; Lee et al., 2009; Chu et al., 2010), a proteomics study of Arabidopsis vacuoles identified AtYSL4 and AtYSL6 in the tonoplast proteome (Jaquinod et al., 2007). To investigate this, we used Arabidopsis protoplasts to transiently express GFP-tagged AtYSL4 and AtYSL6. When protoplasts were transformed with either AtYSL4 or AtYSL6 tagged with GFP at the C-terminus, very few cells became labeled. Indeed, we were unable to observe fluorescence signals in the case of AtYSL6. In the small number of YSL4-GFP transformants identified, we observed fluorescent label accumulating within the ER (data not shown). Based on these findings, we tentatively concluded that end-labeled AtYSL4 and AtYSL6 were being abnormally processed, and we constructed versions of AtYSL4 and AtYSL6 that contain GFP labels in non-conserved regions within each protein (AtYSL4-GFPmid and AtYSL6-GFPmid). We then co-transformed protoplasts with either *AtYSL4-GFPmid* or *AtYSL6-GFPmid* in addition to γ*-TIP-mCherry*, which served as a vacuolar marker. In a separate experiment, we stained protoplasts transformed with *AtYSL6-GFPmid* with FM-464 to provide a marker for internal membranes. It was evident that GFPmid-tagged AtYSL4 localized to the tonoplast membrane, based on colocalization with γ-TIP-mCherry (Figure 4, **Top row**). Additionally, AtYSL6 also localized to the tonoplast membrane, based on colocalization with γ-TIP-mCherry and FM-464 (Figure 4, **middle and bottom rows**, respectively).

**Figure 5:**
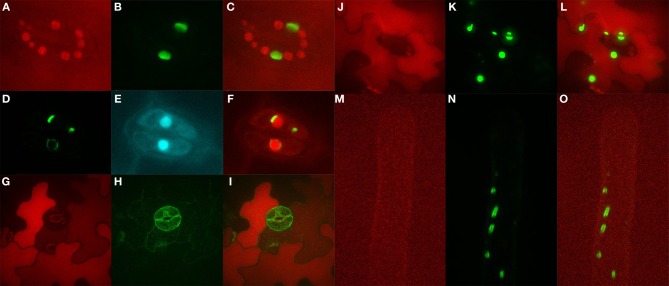
**Subcellular localization of AtYSL6 in stably transformed plants**. The AtYSL6-GFPmid construct, controlled by a ubiquitin promoter, was stably transformed into Arabidopsis plants. **(A)** Red fluorescence of guard cell chloroplasts. **(B)** Green fluorescence in guard cells from **(A)**. **(C)** Overlay of **(A,B). (D)** Green fluorescence in guard cells. **(E)** DAPI fluorescence in guard cells from **(D)**. **(F)** Overlay of **(D,E)**. **(G)** Red autofluorescence in leaf epidermis and guard cells. **(H)** Green fluorescence in cells from **(G)**. **(I)** Overlay of **(G,H)**. **(J)** Red autofluorescence in leaf epidermis and guard cells. **(K)** Green fluorescence in cells from **(J). (L)** Overlay of **(J,K)**. **(M)** Red autofluorescence in a root hair. **(N)** Green fluorescence in root hair from **(M)**. **(O)** Overlay of **(M,N)**.

The *AtYSL6-GFPmid* construct was also used to stably transform Arabidopsis plants. The resulting transgenic plants were morphologically normal, indicating that overexpression of this membrane protein did not cause serious problems with the endomembrane system. In the stable transformants, the GFP signal was most readily observed in the guard cells (Figures 5A–I) Notably, not every cell contained fluorescent material, in spite of the fact that the *AtYSL6-GFPmid* construct was driven by a constitutive promoter. Most often, the signal appeared as a bright spot in each guard cell that did not coincide with the chlorplasts (Figures 5A–C). To identify the bright spots, which are positioned similarly to guard cell nuclei, we used DAPI stain (Figures 5D–F). From this analysis we observed that the green fluorescence was positioned around the nuclei in the guard cells. The signal is not coming from the nuclear envelope, since often it does not completely surround the nucleus. Probably the signal emanates from the endoplasmic reticulum (ER) surrounding nuclei. Another commonly observed pattern of fluorescence was more diffuse staining of internal membranous networks that may be ER (Figures 5G–I). Finally, we often observed fluorescence in bright bodies similar to the small vacuoles that we had observed in protoplasts. These bright bodies were observed in pavement (leaf epidermal) cells (Figures 5J–L), in root cells (not shown) and in root hairs (Figures 5M–O) Consistent with our observations in transiently transformed protoplasts, we never observed fluorescence associated with the large central vacuole in any cell.

### Characterization of *ysl4* and *ysl6* mutant alleles

**Figure 6:**
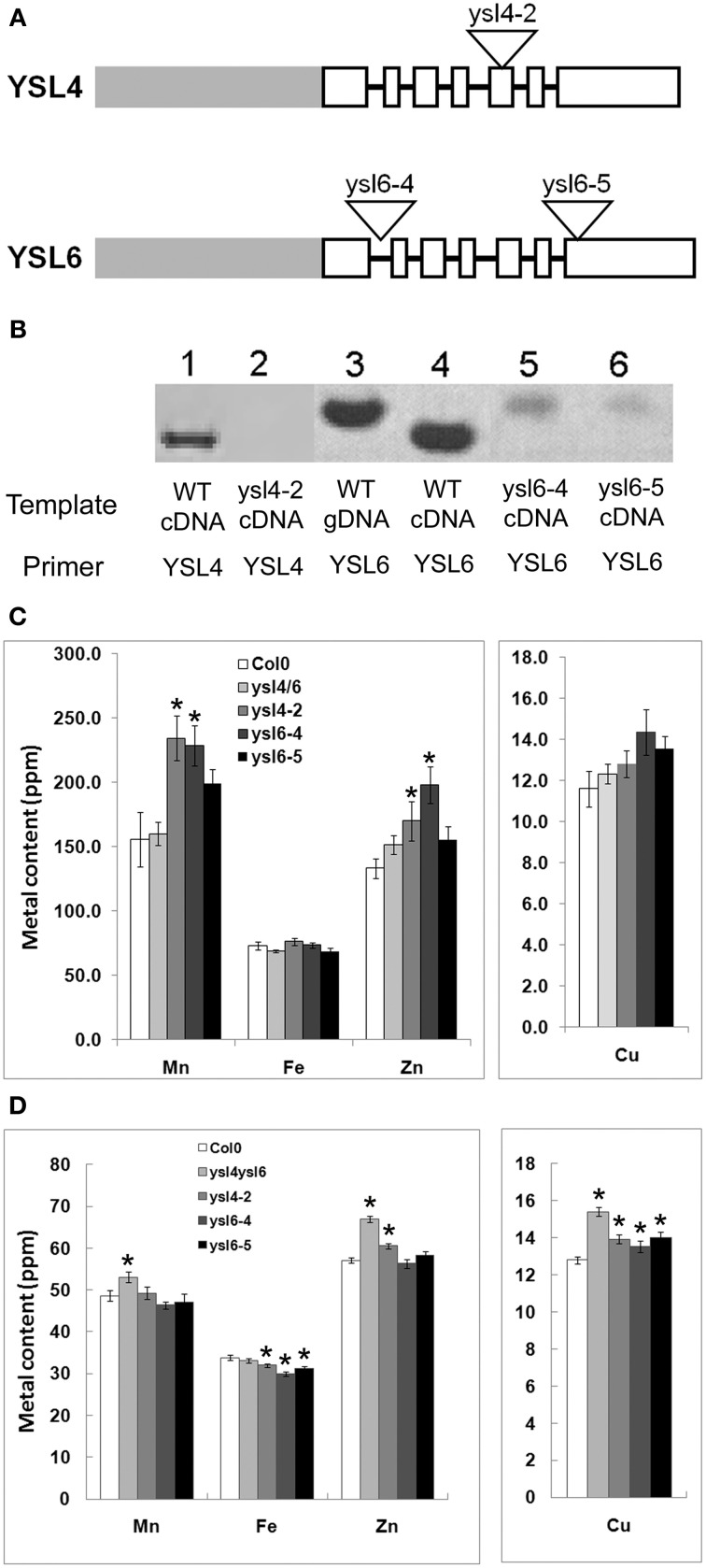
**ysl4-2, ysl6-4 and ysl6-5 null mutants. (A)** Schematic representation of the structure of AtYSL4 and AtYSL6. Gray bars represent promoter regions. White boxes represent exons. Black lines represent introns (not to scale). Triangles represent insertion sites of T-DNAs in the Salk T-DNA insertion mutants, ysl4-2 (SALK_025447), ysl6-4 (SALK_119560), and ysl6-5 (SALK_093392). **(B)** Detection of AtYSL4 and AtYSL6 mRNA. RT-PCR was performed using RNA extracted from the leaves of wild type (WT), ysl4-2, ysl6-4, ysl6-5, and ysl4ysl6 plants. WT genomic DNA (gDNA) was also included as a control. Lane 1: WT cDNA with AtYSL4 specific primers. Lane 2: ysl4-2 cDNA with AtYSL4 specific primers. Lane 3: WT gDNA with AtYSL6 specific primers. Lane 4: WT cDNA with YSL6 specific primers. Lane 5: ysl6-4 cDNA with YSL6 specific primers. Lane 6: ysl6-5 cDNA with AtYSL6 specific primers. **(C)** and **(D)** ICP-MS determination of metal concentrations of Col-0, ysl4-2, ysl6-4, ysl6-5, and ysl4ysl6. Results are given as ppm. Error bars represent standard error. Each sample contains 10 replicates. Asterisks indicate *P* < 0.05 by *t*-test. **(C)** Metal concentrations of leaves. **(D)** Metal concentrations of seeds.

In order to understand the *in planta* functions of AtYSL4 and AtYSL6, we obtained mutant alleles from the SALK collection of sequence-indexed T-DNA insertions (Alonso et al., 2003). A single insertion line (SALK_025447; *ysl4-2*) was confirmed for AtYSL4, in which the T-DNA is inserted in the fifth exon (Figure 6A). The line SALK_006995 is annotated as an insertion into *AtYSL4*, but we were not able to amplify flanking sequences from this line, and thus concluded that the line is likely mis-annotated. Two alleles were identified with T-DNA insertions in *AtYSL6* (SALK_119560; *ysl6-4* and SALK_093392; *ysl6-5*). These insertions were in the first intron and last exon, respectively (Figure 6A). To determine whether these T-DNA insertions caused loss of function mutations, RT-PCR was performed on plants homozygous for each allele (Figure 6B). No *AtYSL4* mRNA was detected in the leaves of *ysl4-2* plants, and no *AtYSL6* mRNA was detected in the leaves of *ysl6-4* and *ysl6-5* plants, although small amounts of contaminating gDNA were detected in these samples. Thus, each of these T-DNA insertions appears to have caused a null mutation.

We determined the metal levels of each single mutant and the *ysl4ysl6* double mutants using ICP-MS (Figures 6C,D). In leaves of plants grown in soil, some statistically significant differences in metal levels were observed, but these did not form a clear pattern. For example, *ysl6-4* mutants had elevated levels of Mn and Zn, yet *ysl6-5* mutants had no significant differences from WT Col-0 plants. Homozygous *ysl4-2* plants also had high Mn and Zn in leaves. Interestingly, however, the *ysl4ysl6* double mutant plants had normal levels of all four metals (Mn, Fe, Zn, and Cu) in leaves.

In the seeds of the mutant plants, more consistent changes in metal levels were observed (Figure 6D). The *ysl4-2* mutant seeds had elevated levels of Zn and Cu and a decreased level of Fe. Both *ysl6-4* and *ysl6-5* mutant seeds had low Fe and elevated Cu. In the *ysl4ysl6* double mutant seeds, Zn and Cu were higher than normal, similar to the *ysl4* and *ysl6* single mutants. However, Fe levels in the seeds of the *ysl4ysl6* double mutants were not significantly different from WT Col-0 despite the single mutants' low seed Fe. The double mutants had elevated Mn levels, which were also not observed in any of the single mutants. The altered metal accumulation phenotypes observed in these mutants suggest that, as expected, AtYSL4 and AtYSL6 play roles in metal ion homeostasis in Arabidopsis.

**Figure 7:**
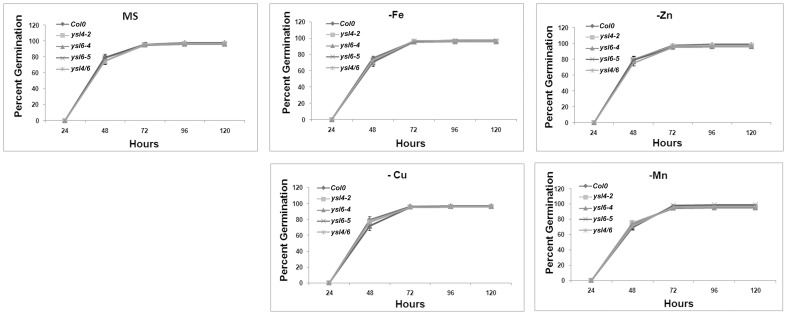
**Germination response to metal deficiency**. Seeds of ysl6-4, ysl6-5, ysl4-2 or the ysl4ysl6 double mutant were germinated on complete MS medium or MS medium lacking Fe, Zn, Cu, or Mn. Germination (scored as emergence of the radicle) was scored every 24 h. Three replicates of 100 seeds each were scored. Error bars indicate standard error of the mean.

**Figure 8:**
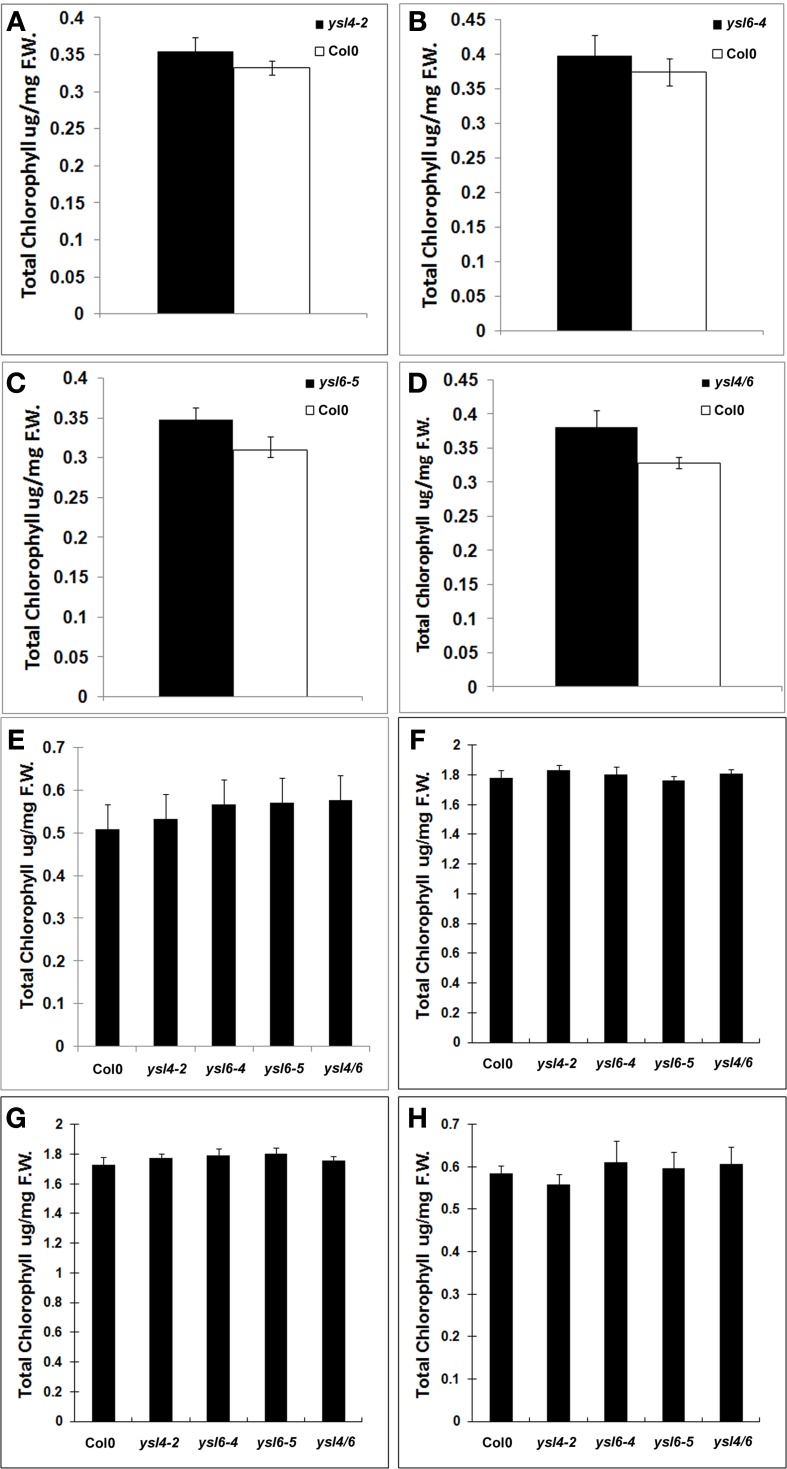
**Metal starvation response in seedlings of Col-0, ysl4-2, ysl6-4, ysl6-5, and ysl4ysl6. (A–D)** Seedlings were grown on MS medium lacking Fe for 5 days and then chlorophyll levels were measured. **(A)** Chlorophyll levels of Col-0 and ysl4-2. **(B)** Chlorophyll levels of Col-0 and ysl6-4. **(C)** Chlorophyll levels of Col-0 and ysl6-5. **(D)** Chlorophyll levels of Col-0 and ysl4ysl6. **(E–H)** Plants were grown on MS plates for 10 days, and then transferred to MS without Fe, Zn, Cu, or Mn for 14 days. The total chlorophyll content of the shoot system was measured. **(E)** Chlorophyll levels of plants grown on MS medium lacking Fe. **(F)** Chlorophyll levels of plants grown on MS medium lacking Zn. **(G)** Chlorophyll levels of plants grown on MS medium lacking Cu. **(H)** Chlorophyll levels of plants grown on MS medium lacking Mn.

No obvious growth defects were noted in the single mutants when grown in soil or on MS agar plates (data not shown), so we identified double mutants that were homozygous for both *ysl4-2* and *ysl6-5*. Like the single mutants, the *ysl4ysl6* double mutants had no obvious growth defects when grown in soil or on MS agar plates (data not shown). We then tested for differential tolerance or sensitivity to metal deficiency in plants either germinated directly on plates lacking Fe, Cu, Mn, or Zn (Figure 7), or in seedlings germinated on plates with normal nutrients, and then transferred to plates lacking one metal (Figure 8). No differences in growth or appearance were identified.

**Figure 9:**
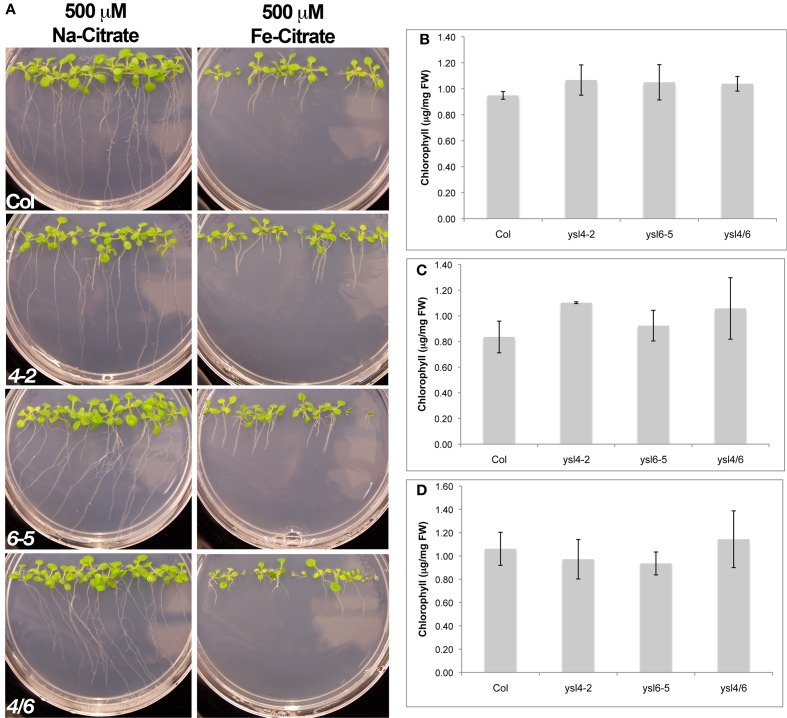
**Response of Col-0, ysl4-2, ysl6-5 and ysl4ysl6 to Fe-citrate excess. (A)** Plants were grown vertically for two weeks on medium containing either 500 mM Na-Citrate or 500 mM FeCitrate prior to photographing. **(B)** Plants were grown for 2 weeks on regular MS medium (no additives, pH 5.7) before measuring chlorophyll content. **(C)** Plants were grown for 2 weeks on MS medium containing 500 mM NaCitrate (pH 5.7) before measuring chlorophyll content. **(D)** Plants were grown for 2 weeks on MS medium that was adjusted to pH 5.7 after addition of 500 mM Fe-Citrate. Chlorophyll content was measured as in **(B,C)**. For **(B–D)**, chlorophyll content of three batches of seedlings was measured and averaged. Error bars indicate ±SD.

We next examined whether *ysl4-2*, *ysl6-5* and *ysl4ysl6* mutants were differentially affected by high levels of iron in the growth medium. Seeds were plated directly onto medium containing either no additional Fe, 500 μM Fe-citrate, or 500 μM Na-citrate. Our initial experiment indicated that, although single mutants were not affected, *ysl4ysl6* double mutants were more sensitive to 500 μM Fe-citrate based on chlorosis and smaller seedling size (Figure 9A). This is finding is consistent with the results presented recently by Divol et al. (Divol et al., 2013). However, we discovered that the addition of 500 μM Fe-citrate caused the pH of the medium to decrease from 5.7–4.0. We thus set up an additional experiment in which we buffered the growth medium such that plates containing 500 μM Fe-citrate remained at pH 5.7. After 2 weeks of growth, we did not observe any differences in seedling appearance on these plates, and there were no measurable differences in chlorophyll content (Figures 9B–D). Thus, without the lowered pH brought about by the inclusion of high concentrations of Fe-citrate in the medium, the double mutant plants were not unusually sensitive to excess Fe.

### Localization of metals in *ysl4* and *ysl6* mutant seeds

**Figure 10:**
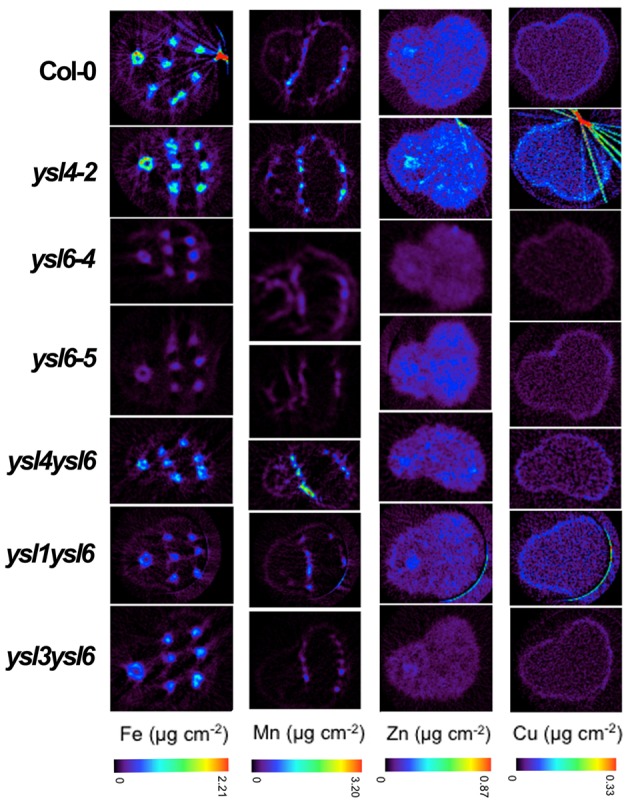
**Synchrotron X-ray Fluorescence (SXRF) Computed Microtomography showing the distribution of Fe, Mn, Zn, and Cu in Col-0 and ysl mutant lines**. Tomograms (virtual cross sections) were collected via SXRF computed microtomography from intact, dry Arabidopsis seed. All seeds are oriented with the radicle at left and cotyledons at right.

Because altered seed metal levels were observed using ICP-MS, we examined whether metals were also mis-localized in the seeds of the mutants. We used synchrotron x-ray fluorescence microtomography (SXFM) to visualize metals directly in the seeds of *ysl4-2*, *ysl6-4*, *ysl6-5* and the double mutant *ysl4ysl6* (Figure 10). Fe localizes to the provascular strands of the hypocotyl, radicle and cotyledons; Mn to the abaxial (lower) epidermis of the cotyledons; and Zn and Cu localize throughout the embryo in a diffuse pattern (Kim et al., 2006). The patterns of metal localization in the single and double mutants were unaltered, indicating that AtYSL4 and AtYSL6 are not required for proper localization of metals in Arabidopsis seeds, even though the levels of these metals are altered in the mutants (Figure 6D).

### Interaction of AtYSL4 and AtYSL6 with AtYSL1 and AtYSL3

It has been established that loss of AtYSL1 and AtYSL3 causes altered metal accumulation in the seeds (Waters et al., 2006; Chu et al., 2010). Because the weak phenotypes of the *ysl4*, *ysl6*, and *ysl4ysl6* mutants were difficult to interpret, we constructed double mutants of *ysl1ysl4*, *ysl1ysl6* and *ysl3ysl6*. Previously, we have shown that *ysl1ysl3* double mutants have severe developmental defects that include chlorosis, male infertility, impaired or aborted seed development, and altered levels of metals in both vegetative structures and seeds (Waters et al., 2006; Chu et al., 2010). Single *ysl1* mutants have mild phenotypes that include low iron in seeds and elevated NA levels (Le Jean et al., 2005), while single *ysl3* mutants are not distinguishable from WT plants. Neither *ysl1ysl4*, *ysl1ysl6* nor *ysl3ysl6* plants displayed strong phenotypes either on soil or MS agar plates (data not shown). Plants had normal chlorophyll levels and normal fertility (data not shown). Additionally, metal localization was not disrupted in either the *ysl1ysl6* or the *ysl3ysl6* double mutant lines based on SXFM experiments (Figure 10).

### Single mutants *ysl4-2*, *ysl6-4*, *ysl6-5* and the double mutant *ysl4ysl6* are sensitive to high levels of manganese

**Figure 11:**
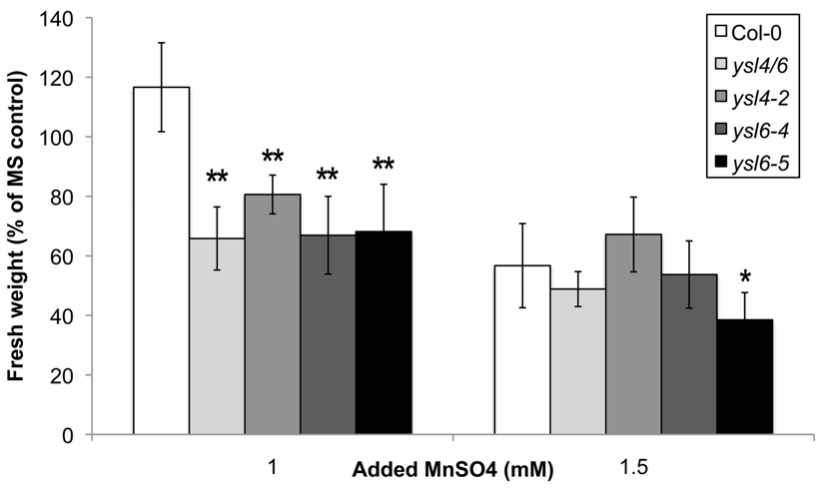
**Manganese sensitivity of *ysl* mutants**. Seeds of Col-0, *ysl4ysl6* double mutants, *ysl4-2*, *ysl6-4* and *ysl6-5* single mutants were sown onto MS medium containing either 0, 1.0 or 1.5 mM excess MnSO4 and grown for 21 days. Fresh weights were measured by weighing batches of 5 seedlings each. Weights are expressed as a percentage of the weights of seedlings grown under the control condition (MS medium without excess MnSO4). *n* = 3–5 batches of seedlings. ^*^p ≤ 0.05 and > 0.01, ^**^p ≤ 0.01.

**Figure 12:**
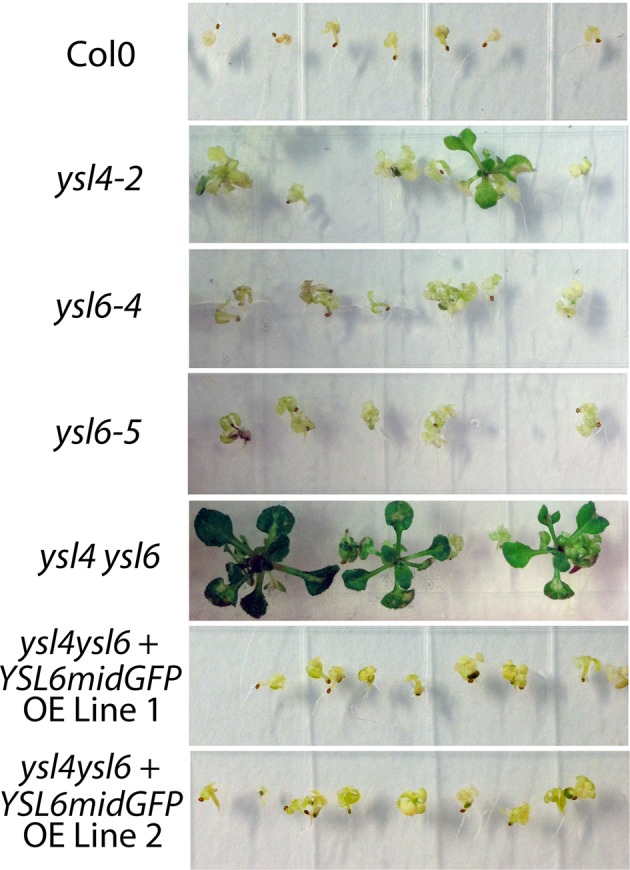
**Prolonged growth of mutants and YSL6-midGFP-OE complemented mutants on excess Mn**. Seeds of Col0, *ysl4-2*, *ysl6-4* and *ysl6-5* single mutants, *ysl4ysl6* double mutants and two independent lines of *ysl4ysl6* double mutants transformed with a construct over expressing the AtYSL6midGFPconstruct (YSL6midGFP) were sown onto 1/2X MS medium containing 1.0 mM excess MnSO4 and grown for 35 days.

Recently, Sasaki *et al.* uncovered a Mn-sensitivity phenotype in rice *OsYSL6* knockout lines. This finding prompted us to examine *AtYSL4* and *AtYSL6* mutants for manganese sensitivity phenotypes. After 21 days of growth, we measured the fresh weights of mutant and wild-type plants grown on MS agar plates containing 0, 1, and 1.5 mM additional Mn. We found that at 1 mM additional Mn, *ysl4-2*, *ysl6-4*, *ysl6-5* and *ysl4ysl6* double mutants had significant decreases in fresh weight compared to Col-0 (Figure 11). At 1.5 mM additional Mn, all plants were severely affected, with only *ysl6-5* showing a significantly lower fresh weight compared to Col-0. When the plants grown for an additional 2 weeks on 1/2X MS plates supplemented with 1 mM Mn, the double mutant plants became green, and grew larger than either single mutants or WT Col0 (Figure 12). Double mutant plants transformed with the *YSL6midGFP* construct did not show growth recovery, but remained small and yellow on 1 mM Mn (Figure 12). Since the *YSL6midGFP* construct restores a WT phenotype (poor growth during prolonged exposure to 1 mM Mn), it appears to be functional *in vivo*.

**Figure 13:**
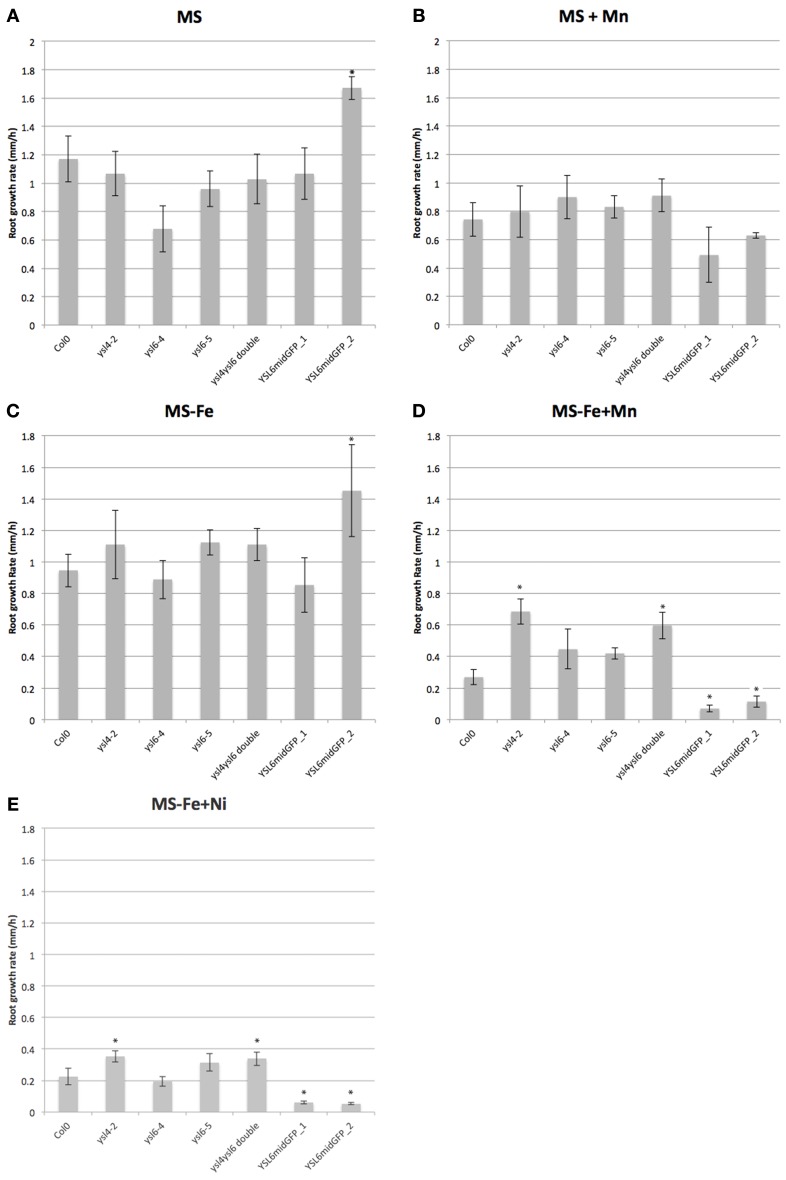
**Growth of mutants and YSL6-midGFP-OE plants on excess metals**. Root growth rate during the 24 h period between 24 h and 48 h post-germination is presented. Photographs of the plates were taken every 24 h, and used to determine the growth rate for each 24 h period (see Materials and Methods). Plants were germinated and grown vertically on plates containing. **(A)** 1/2X MS (MS). **(B)** 1/2X MS + 1 mM MnSO4 (MS + Mn). **(C)** 1/2X MS without added iron (MS-Fe). **(D)** 1/2X MS without added iron and with 1 mM MnSO4 (MS−Fe+Mn). **(E)** 1/2X MS without added iron and with 90 uM NiCl2 (MS−Fe+Ni). ^*^p ≤0.05.

We also measured the rate of root growth in early seedlings exposed to 1 mM MnSO_4_, as well as 90 um NiCl_2_ and 500 uM ZnSO_4_, to see whether mutation of *YSL4* and *YSL6* or over-expression of *YSL6midGFP* would affect the plants' ability to grow in the presence of toxic levels of these metals (Figure 13). To force the plants to take up excess Mn, Zn, or Ni, we included plates that were prepared with no iron. Under these iron deficient conditions, plants are expected to up-regulate *IRT1* expression, which leads to increase uptake of iron, and of other IRT1 substrates like Mn, Ni and Zn (Baxter et al., 2008).

One of the two lines of plants overexpressing YSL6midGFP had an increased root growth rate on 1/2MS medium (Figure 13A). On 1/2X MS containing added Mn, there was a trend (Figure 13B; not statistically significant) of decreased root growth rate for the YSL6midGFP plants, but no trends or significant differences were noted on 1/2XMS containing added Ni (not shown). When iron was withdrawn from the medium, however, both mutants and over-expressing plants showed marked changes in root growth rates (Figures 13D,E). In the presence of either Mn or Ni, the *ysl4* mutants and the *ysl4ysl6* double mutants had increased root growth rates, while *AtYSL6-GFPmid* overexpressing plants had decreased root growth rates. We did not observe any significant differences in the growth rates of mutant or over-expressing plants exposed to 500 uM Zn (not shown).

#### Transport of metals by AtYSL4 and AtYSL6

**Figure 14:**
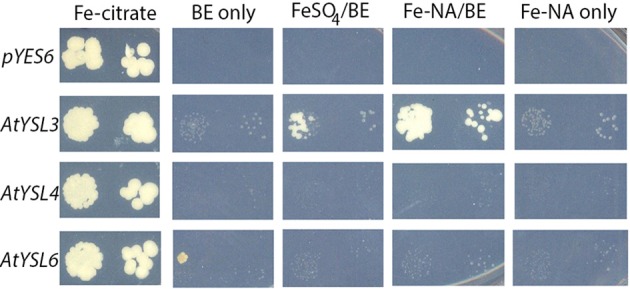
**Neither *AtYSL4* nor *AtYSL6* is able to complement *fet3fet4***. These experiments were performed as described (Chu et al. 2010). *fet3fet4* yeast (derived from DEY1453) transformed with *pGEV* and *pYES6/CT* empty vector (top row) or constructs containing *AtYSL3* (middle row), *AtYSL4* (third row), and AtYSL6 (bottom row) were plated onto SD-TRP medium containing 50 mM Fe-Citrate (first column), 0 mM iron + 10 nM beta-estradiol (BE) (second column), 5 mM FeSO4 + 10 nM BE (third column), 5 mM Fe-NA complex + 10 nM BE (fourth column), or 5 mM Fe-NA complex without BE (last column). Pairs of spots correspond to 100 and 1000 fold dilutions of the original cultures. Plates were photographed after 10 days of growth.

We tested transporter activity of AtYSL4 and AtYSL6 using yeast functional complementation assays, but neither protein could alleviate the iron-limited growth defect of *fet3fet4* yeast (Figure 14). Because successful complementation will only occur if heterologous proteins are expressed on the yeast plasma membrane, it is reasonable that vacuolar proteins AtYSL4 and AtYSL6 would not correct the *fet3fet4* growth defect. Indeed, HvYSL5, which belongs to the same group as AtYSL4 and AtYSL6 and was shown to localize to vesicles in barley cells, was unable to complement *fet3fet4* yeast in the presence of 20 mM Fe-NA (Zheng et al., 2011). The use of other transport assay systems will be required in order to characterize the transport activity of these proteins.

## Discussion

### AtySL4 and AtYSL6 are associated with internal membranes and small vacuoles

In this paper, we provide evidence to indicate that two members of the Arabidopsis YSL family, AtYSL4 and AtYSL6, function at internal membranes. All previously characterized YSL proteins are located on the plasma membrane (DiDonato et al., 2004; Aoyama et al., 2009; Inoue et al., 2009; Lee et al., 2009; Chu et al., 2010) or on PM associated vesicles (Zheng et al., 2011). Interestingly, the localization pattern of OsYSL6, which belongs to the same group as AtYSL4 and AtYSL6, could not be definitely determined. In transient transformation experiments, the GFP signal accumulated throughout the cell regardless of whether OsYSL6 was tagged at the C-terminus or the N-terminus (Sasaki et al., 2011). Using GFPmid tagged proteins, which have GFP inserted in non-conserved central region of each protein, we have shown that AtYSL4-GFPmid and AtYSL6-GFPmid localize to the vacuolar membrane based on co-localization with γ-TIP-mCherry. Additionally, AtYSL6-GFPmid co-localized with FM-464, a lipophilic membrane stain that can efficiently label vacuolar membranes (Kutsuna and Hasezawa, 2002). In stably transformed plants, fluorescence from the *AtYSL6-GFPmid* construct was also observed on diffuse internal membranes, possibly the ER. The *AtYSL6-GFPmid* construct appears to be functional, since stably transformed plants that overexpress this construct do not have growth defects under normal growth conditions, but do show distinct metal-related phenotypes.

Vacuoles are an important site for storage of metals, with more than 90% of the zinc in the cell and approximately 50% of the iron in the cell sequestered in the vacuoles (Lanquar et al., 2010). In Arabidopsis, the vacuolar transporter MTP1 is required for plant responses to Zn excess, implying that vacuolar sequestration of Zn is critical for preventing cellular damage caused by excess Zn (Kobae et al., 2004; Desbrosses-Fonrouge et al., 2005). The vacuole also serves as an important storage place for iron during embryo development. In stage VI wild type embryos, the main pool of Fe is held in the vacuoles of cells surrounding the pro-vascular system (Kim et al., 2006). Loss of the vacuolar iron importer VIT1 caused a redistribution of iron to a single sub-epidermal cell layer in the cotyledon, although the iron remained in vacuoles (Roschzttardtz et al., 2009). This finding suggests the existence of other vacuolar Fe import systems. AtNRAMP4, which is known to be involved in remobilization of vacuolar Fe during germination (Lanquar et al., 2005), co-localizes with γ-TIP (Bolte et al., 2011). γ-TIP has recently been shown to label structures embedded inside the protein storage vacuole (PSV) of dry Arabidopsis seeds(Bolte et al., 2011), and γ-TIP has been found associated with globoid structures in dry seeds of tobacco (Jiang et al., 2001). Because AtYSL4 and AtYSL6 also co-localize with γ-TIP, it is possible that they also function within PSVs. The Metal Tolerance Proteins, MTP1 and MTP3 are responsible for transporting zinc into vacuoles (Desbrosses-Fonrouge et al., 2005; Arrivault et al., 2006). Recently ZIF1 was identified as a transporter that can move NA into vacuoles. Thus, metal NA complexes are expected to occur in vacuoles, and the existence of tonoplast transporters capable of moving metal-NA complexes across this membrane is logical.

Vacuolar sequestration or excess heavy metals is particularly important during iron deficiency, when increased activity of the IRT1 transporter causes excessive uptake of Mn, Ni, and Zn, as well as some other heavy metals, if they are present in the growth medium(Eide et al., 1996; Korshunova et al., 1999; Baxter et al., 2008) In Arabidopsis, the tonoplast-localized Metal Transport Protein3 (MTP3) is critical for moving excess Zn accumulated during iron deficiency into the vacuole (Arrivault et al., 2006). MTP3 is positively regulated by iron deficiency, and also by excess Zn and Co. In the absence of MTP3, plants grown under Fe deficiency are unable to sequester Zn in the roots, resulting in increased Zn accumulation in the shoots.

Metal transporters are also found in the endomembrane system, where they are responsible for providing metals to metalloproteins located in the ER and/or Golgi. For example, the copper transporter RAN1 is required for biogenesis of ethylene receptors (Binder et al., 2010) and the probable zinc transporter IAR1 is required for the activity of ER localized IAA-amino acid conjugate hydrolases(Lasswell et al., 2000). The Golgi-localized P-type ATPase, ECA3, is required for growth under Mn deficiency, while the prevacuolar compartment (PVC) localized manganese transporter MTP11 is required for maintaining correct levels of Mn in tissues, and for growth on excess Mn (Delhaize et al., 2007).

The model best supported by the data presented here is that AtYSL4 and AtYSL6 participate in the provision of Mn and Ni to proteins located in internal cellular compartments. Loss of *YSL4* and *YSL6* function allowed improved long term development of shoots grown on excess Mn (Figure 12), and improved growth of roots exposed to excess Mn and excess Ni (Figure 13), while over-expression of *YSL6* caused decreased growth of roots on excess Mn or Ni. This suggests that, when Mn or Ni are plentiful in the cytoplasm, these YSLs promote transport of excess metals into intracellular compartments, causing impaired growth. If the role of the YSLs were in Mn or Zn sequestration (similar to the role of MTP3), we would expect the opposite phenotype: *ysl4* and *ysl6* mutants would be expected to have diminished growth in conditions of metal excess, and overexpressors would be expected to have better growth in metal excess.

In a recent publication, Divol et al. (2013) raised polyclonal antibodies against AtYSL6, and used these in immunofluorescence microscopy to conclude that AtYSL6 is localized to plastids. In our experiments using GFP-tagged AtYSL6, we never observed green fluorescence signals associated with chloroplasts. Instead, we observed fluorescence signals on the vacuole membrane, and on other, undefined internal membranes in stably transformed plants. The *YSL6midGFP* construct used in our studies appears to be functional, since it complements the phenotype of the *ysl4ysl6* double mutant plants, conferring poor growth during prolonged exposure to 1 mM Mn. In these stably transformed plants, we often observed chloroplasts, which have strong red autofluorescence, but never observed any indication of green GFP fluorescence signal (see, for example, Figure 5). Additional work will be needed to resolve why the different techniques used for localization of AtYSL4 and AtYSL6 showed such marked differences.

It is interesting to note Divol et al.'s examination of vacuolar iron using Perl's/DAB staining in 2D imbibed embryos. In these embryos, Fe mobilization from vacuolar storage has commenced. *ysl4ysl6* double mutants have higher than normal vacuolar Fe while YSL6 over-expressing plants have lower than normal vacuolar Fe during this time(Divol et al., 2013). This pattern is consistent with YSL4 and YSL6 as vacuolar effluxers of iron (or Fe-NA complexes) during germination. We note, however, that no consistent germination effects in the *ysl4*, *ysl6* or *ysl4ysl6* double mutants were noted in spite of extensive investigation during the course of our studies (Figure 10, and data not shown).

### Transport of metals by AtYSl4 and AtYSL6

Several lines of evidence indicate that AtYSL4 and AtYSL6 function in metal ion transport. First, mutants (either single or double) have altered metal accumulation patterns in shoots or in seeds. This suggests that, like other YSL proteins, AtYSL4 and AtYSL6 transport metals. *ysl4* and *ysl6* single mutants, along with the *ysl4ysl6* double mutant, are sensitive to high levels of manganese. Under conditions that increase the expression of the promiscuous transporter IRT1, both mutants and overexpressing plants exhibit altered rates of root elongation. Mutant plants (*ysl4* and *ysl4ysl6* double mutants) display increased root growth under these conditions, while YSL6-GFPmid overexpressing plants have decreased root elongation under these conditions. Indeed, when single (*ysl4-2, ysl6-4*, or *ysl6-5*) or double mutants are grown for 21 days on 1 mM Mn, they accumulate less shoot mass. This, combined with their vacuolar/endomembrane system localization patterns, suggests a role for AtYSL4 and AtYSL6 in sequestration or release of heavy metals such as manganese and nickel from the plant endomembrane system and vacuole.

Divol et al. (2013) reported that *ysl4ysl6* double mutants are sensitive to excess Fe in the growth medium. We also observed sensitivity of the double mutant to Fe-Citrate, but show here that 500 uM Fe-citrate used in the experiment also causes a dramatic lowering of the pH of the medium. When pH of the medium is buffered to 5.7, as is usual for Arabidopsis growth, the sensitivity phenotype of the double mutant was not observed. Since Fe solubility is strongly affected by pH, the amount of soluble iron is likely higher on the un-buffered plates than the buffered ones, making it difficult to experimentally separate the effect of Fe vs. the effect of pH. Thus, while YSL4 and YSL6 may have roles in internal Fe transport, experimental evidence for this is not completely clear. Direct measurement of transport activity, which has not been achieved yet, would help to elucidate this point.

### Conflict of interest statement

The authors declare that the research was conducted in the absence of any commercial or financial relationships that could be construed as a potential conflict of interest.
